# Zagreb Amblyopia Preschool Screening Study: near and distance visual acuity testing increase the diagnostic accuracy of screening for amblyopia

**DOI:** 10.3325/cmj.2016.57.29

**Published:** 2016-02

**Authors:** Mladen Bušić, Mirjana Bjeloš, Mladen Petrovečki, Biljana Kuzmanović Elabjer, Damir Bosnar, Senad Ramić, Daliborka Miletić, Lidija Andrijašević, Edita Kondža Krstonijević, Vid Jakovljević, Ana Bišćan Tvrdi, Jurica Predović, Antonio Kokot, Filip Bišćan, Mirna Kovačević Ljubić, Ranka Motušić Aras

**Affiliations:** 1 University Eye Clinic, University Hospital “Sveti Duh,” Zagreb, Croatia; 2 Faculty of Medicine, Josip Juraj Strossmayer University of Osijek, Osijek, Croatia; 3 Dubrava University Hospital, Zagreb, Croatia; 4 Department of Medical Informatics, Rijeka University School of Medicine, Rijeka, Croatia; 5 Polyclinic “Ghetaldus,” Zagreb, Croatia; 6 Polyclinic “Medikol,” Zagreb, Croatia; 7 Department of Anatomy and Neuroscience, Faculty of Medicine, Josip Juraj Strossmayer University of Osijek, Osijek, Croatia; 8 Department of Ophthalmology, General Hospital Zabok, Zabok, Croatia

## Abstract

**Aim:**

To present and evaluate a new screening protocol for amblyopia in preschool children.

**Methods:**

Zagreb Amblyopia Preschool Screening (ZAPS) study protocol performed screening for amblyopia by near and distance visual acuity (VA) testing of 15 648 children aged 48-54 months attending kindergartens in the City of Zagreb County between September 2011 and June 2014 using Lea Symbols in lines test. If VA in either eye was >0.1 logMAR, the child was re-tested, if failed at re-test, the child was referred to comprehensive eye examination at the Eye Clinic.

**Results:**

78.04% of children passed the screening test. Estimated prevalence of amblyopia was 8.08%. Testability, sensitivity, and specificity of the ZAPS study protocol were 99.19%, 100.00%, and 96.68% respectively.

**Conclusion:**

The ZAPS study used the most discriminative VA test with optotypes in lines as they do not underestimate amblyopia. The estimated prevalence of amblyopia was considerably higher than reported elsewhere. To the best of our knowledge, the ZAPS study protocol reached the highest sensitivity and specificity when evaluating diagnostic accuracy of VA tests for screening. The pass level defined at ≤0.1 logMAR for 4-year-old children, using Lea Symbols in lines missed no amblyopia cases, advocating that both near and distance VA testing should be performed when screening for amblyopia.

Vision disorders in children represent important public health concern as they are acknowledged to be the leading cause of handicapping conditions in childhood (1). Amblyopia, a loss of visual acuity (VA) in one or both eyes (2) not immediately restored by refractive correction (3), is the most prevalent vision disorder in preschool population (4). The estimated prevalence of amblyopia among preschool children varies from 0.3% (4) to 5% (5). In addition, consequences of amblyopia include reduced contrast sensitivity and/or positional disorder (6). It develops due to abnormal binocular interaction and foveal pattern vision deprivation or a combination of both factors during a sensitive period of visual cortex development (7). Traversing through adulthood, it stands for the leading cause of monocular blindness in the 20-70 year age group (8). The main characteristic of amblyopia is crowding or spatial interference, referring to better VA when single optotypes are used compared to a line of optotypes, where objects surrounding the target object deliver a jumbled percept (9-12). Acuity is limited by letter size, crowding is limited by spacing, not size (12).

Since amblyopia is predominantly defined as subnormal VA, a reliable instrument for detecting amblyopia is VA testing (13-15). Moreover, VA testing detects 97% of all ocular anomalies (13). The gold standard for diagnosing amblyopia is complete ophthalmological examination (4). There is a large body of evidence supporting the rationale for screening, as early treatment of amblyopia during the child’s first 5-7 years of life (8) is highly effective in habilitation of VA, while the treatment itself is among the most cost-effective interventions in ophthalmology (16). Preschool vision screening meets all the World Health Organization’s criteria for evaluation of screening programs (17). Literature search identified no studies reporting unhealthy and damaging effects of screening. The gold standard for screening for amblyopia has not been established (4). There is a large variety of screening methodologies and inconsistent protocols for referral of positives to complete ophthalmological examination. Lack of information on the validity (18,19) and accuracy (4) of such protocols probably intensifies the debate on determining the most effective method of vision screening (8,20-29). The unique definition of amblyopia accepted for research has not reached a consensus (4,5,30,31), further challenging the standardization of the screening protocols.

Overall, two groups of screening methods exist: the traditional approach determines VA using VA tests, while the alternative approach identifies amblyogenic factors (27) based on photoscreening or automated refraction. The major difference between the two is that VA-based testing detects amblyopia directly, providing an explicit measure of visual function, while the latter, seeking for and determining only the level of refractive status does not evaluate visual function. In addition, the diagnosis and treatment of amblyopia is governed by the level of VA. On the other hand, amblyogenic factors represent risk factors for amblyopia to evolve. There are two major pitfalls in screening for amblyogenic factors. First, there is a lack of uniform cut-off values for referral and second, not all amblyogenic factors progress to amblyopia (19).

Besides the issue of what should be detected, amblyopia or amblyogenic factors, a question is raised about who should be screened. Among literate children, both 3- and 4- year-old children can be reliably examined. However, 3-year-old children achieved testability rate of about 80% and positive predictive rate of 58% compared to >90% and 75%, respectively in the 4-year-old group (32). In addition, over-referrals are more common among 3-year-old children (32). These data determine the age of 4 years as the optimum age to screen for amblyopia. Hence, testability is a relevant contributor in designating the optimal screening test.

If VA is to be tested in children, accepted standard tests should be used, with well-defined age-specific VA threshold determining normal monocular VA. For VA testing of preschool children Lea Symbols (33) and HOTV charts (22,32) are acknowledged as the best practice (34), while tumbling E (28,35,36) and Landolt C (28,37-39) are not appropriate as discernment of right-left laterality is still not a fully established skill (34,40). The Allen picture test is not standardized (34,41). Both Lea Symbols and HOTV optotypes can be presented as single optotypes, single optotypes surrounded with four flanking bars, single line of optotypes surrounded with rectangular crowding bars, or in lines of optotypes (22,33,34,41-53). The more the noise, the bigger the “crowding” effect. Isolated single optotypes without crowding overestimate VA (24), hence they are not used in clinical practice in Sweden (32). If presented in lines, which is recognized as the best composition to detect crowding, test charts can be assembled on Snellen or gold standard logMAR principle (34,42,51,54). Age-specific thresholds defining abnormal VA in preschool screening for amblyopia changed over time from <0.8 to <0.65 for four-year-old children due to overload of false positives (20).

The outline of an effective screening test is conclusively demonstrated by both high sensitivity and high specificity. Vision screening tests predominately demonstrated higher specificity (4). Moreover, sensitivity evidently increased with age, whereas specificity remained evenly high (4). The criteria where to set the cut-off point if the confirmatory, diagnostic test is expensive or invasive, advocate to minimize false positives or use a cut-off point with high specificity.

On the contrary, if the penalty for missing a case is high and treatment exists, the test should maximize true positives and use a cut-off point with high sensitivity (55). A screening test for amblyopia should target high sensitivity to identify children with visual impairment, while the specificity should be high enough not to put immense load on pediatric ophthalmologists (14). Complete ophthalmological examination as the diagnostic confirmatory gold standard test for amblyopia is neither invasive nor elaborate technology is needed, while the penalty for missing a case is a lifetime disability.

In devising the Zagreb Amblyopia Preschool Screening (ZAPS) study protocol, we decided to use Lea Symbols in lines test and to screen preschool children aged 48-54 months to address the problems declared. Near VA testing was introduced in addition to commonly accepted distance VA testing (14,22,24,32,45,56-69) due to several reasons: first, hypermetropia is the most common refractive error in preschool children (70), hence near VA should more reliably detect the presence of hypermetropia; second, the larger the distance, the shorter the attention span is; and third, to increase the accuracy of the test.

The pass cut-off level of ≤0.1 logMAR was defined because of particular arguments. Prior to 1992 Sweden used the pass cut-off level for screening of 0.8 (20). A change in the referral criteria to <0.65 for four-year-old children ensued, as many children referred did not require treatment (20). In addition, amblyopia treatment outcome of achieved VA>0.7 is considered as habilitation of normal vision (3,14). At last, the pass cut-off value ≤0.1 logMAR at four years can hardly mask serious visual problems, and even if they are present, we presume they are mild and can be successfully treated at six years when school-entry vision screening is performed. The aim of the ZAPS study is to present and evaluate new screening protocol for preschool children aged 48-54 months, established for testing near and distance VA using Lea Symbols in lines test. Furthermore, we aimed to determine the threshold of age-specific and chart-specific VA normative, testability of the ZAPS study protocol, and the prevalence of amblyopia in the City of Zagreb County. By delivering new evidence on amblyopia screening, guideline criteria defining optimal screening test for amblyopia in preschool children can be revised in favor of better visual impairment clarification.

## Methods

The ZAPS study protocol and informed consent were confirmed by the Institutional Ethics Review Board of the University Hospital “Sveti Duh,” Zagreb. All study procedures adhered to the institutional and governmental legislations regarding ethical principles for medical research involving human subjects, and the tenets of the Declaration of Helsinki. A parent or guardian (hereinafter referred to as “parent“) of each study participant gave written informed consent.

### Study population

The sample size of this cross-sectional, population-based study, consisted of 16 896 eligible children aged 48-54 months attending kindergartens in the City of Zagreb County, in the period September 2011 – June 2014. Parental consent was obtained for 15 648 (92.61%) children, 998 (5.91%) parents did not respond, while 250 (1.48%) parents rejected for their child to participate. Testing was attempted in kindergartens, on all 15 648 children, regardless of any known developmental or visual disability. Children who wore eyeglasses were tested with their eyeglasses (37).

All screeners (N = 17) were attentively and thoroughly trained prior to testing and data collection. Care was taken about equal distribution of probands per screener.

### Study design

The ZAPS study was designed to be performed in four phases. LEA SYMBOLS® in lines Pediatric Eye Near and Distance Charts (Good Lite, Elgin, USA), presenting optotypes in the ETDRS format (51) using logMAR progression to detect crowding, were used to test near (40 cm) and distance (3 m) VA. Phase I, II, and III were carried out in the kindergartens of The City of Zagreb County by general ophthalmologists and residents in ophthalmology. Phase IV was conducted at the University Eye Clinic, University Hospital “Sveti Duh“ in Zagreb by pediatric ophthalmologists.

Phase I included binocular pre-testing to assess whether the child is cognizant with the picture optotypes. In Phase II monocular vision testing was conducted, and if the VA in either eye was >0.1 logMAR, the child was re-tested in Phase III ZAPS study protocol. If failed at re-test, the child was referred to the Eye Clinic for comprehensive eye examination to be performed as the gold standard procedure for diagnosing amblyopia.

Each parent of the eligible child received an in-home brief medical history inquiry including the informed consent form. The inquiry incorporated a brochure with general information on amblyopia, emphasizing the significance of screening along with the subjective, parental-reporting assessment of child’s ocular risk factors, general health, and environment-related status. As soon as inquiries were collected, VA assessment was carried out.

Phase I. Binocular pre-testing was performed using Lea Symbols in lines distance chart at 0.5 m with 30 M letter line to assess whether the child is cognizant with the picture optotypes. Monocular vision testing ensued when the child identified all four optotypes accurately. If the child did not respond at all, the child was labeled as non-cooperative.

Phase II. Monocular vision testing was conducted with the right eye approached first, proceeded to the left, with the non-testing eye occluded with an adhesive patch. The examination was performed at near (40 cm), followed by distance (3 m) VA measurement. Beginning with the 30 M line, the examiner continued through descending lines requesting the child to read a single optotype per line until replied falsely. When incorrect, the examiner presented optotypes two lines above the line failed and asked the child to read the whole line. Four out of five optotypes in a line accurately interpreted constituted the pass criterion for the line. The pass criterion for the first line 30 M was three out of four optotypes. The VA score, measured in logMAR was documented. The pass cut-off level was ≤0.1 logMAR, with fewer than two lines difference between the eyes. If the VA in either eye was >0.1 logMAR, the child was referred to Phase III. A child who had incomplete results in either eye was classified as non-testable and was proceeded to Phase III. The criteria for referral to Phase III regardless of the VA pass were suspected strabismus and ocular disease at examiner’s discretion.

Phase III. The protocol for re-testing the children who failed the Phase I and II, followed the same testing procedure outlined in Phase I and II. The re-testing was performed 1 month after the initial test (Phase II). If the VA in either eye was >0.1 logMAR, the child was referred to the University Eye Clinic to Phase IV. The criteria for referral to complete ophthalmological examination regardless of the VA pass were suspected strabismus and ocular disease, at examiner’s discretion.

Phase IV. The in-clinic protocol for presenting VA testing of referred children followed the Phase I and Phase II testing procedure. If the VA in either eye was >0.1 logMAR or the child was non-testable or non-cooperative, the in-clinic standardized comprehensive eye examination was performed, including the following (27-29,34,37): history taking (date of birth, sex, pregnancy, delivery, and neonatal history, gestational age and maturity, birth weight, disability at birth, congenital anomalies, developmental delay, general health, ocular health and vision disability, history of trauma, family general and eye history); Brückner test (71-75) and Hirschberg test (72,76); Titmus stereoacuity test (77) (Stereo Fly Test®, STEREO OPTICAL CO., INC. Chicago, IL, USA); cover-uncover and alternating cover test for ocular alignment assessment; assessment of motility (versions, ductions, and saccades); pupil and anterior segment examination; cycloplegic retinoscopy with refractive error determination; indirect ophthalmoscopy for fundus examination. The anterior segment was evaluated using a slit lamp. Cycloplegic refraction was performed using 1.0% tropicamide (Mydriacyl® 1%, ALCON – COUVREUR, Puurs, Belgium), at least 15 minutes after instilling the third of three drops, administered in 15-minute intervals. Distance VA was re-tested on the same day with a trial lens spectacle wearing full cycloplegic correction consistent with presenting VA testing protocol (78).

### Definition of amblyopia, refractive error, and testability

The Eye Clinic Expert Panel of Investigators established the criteria for amblyopia, refractive error, and testability derived from consensus, reviews, and evaluation of existing evidence from major vision screening studies (22,27,30,79,80). The criterion for unilateral amblyopia held to ≥2 lines interocular difference (IOD), after re-testing the child on the comprehensive eye examination wearing full cycloplegic correction, with the best corrected VA of >0.1 logMAR in the worse eye presented with amblyogenic factor.

Amblyogenic factors were defined as follows: hyperopia ≥2.00 D spherical equivalent (SE); myopia ≥3.00 D SE; astigmatism at any-axis ≥1.00 D; anisometropia ≥1.00 D difference in hypermetropia, ≥3.00 D difference in myopia, or ≥1.00 D difference in astigmatism in any meridian; antimetropia with ≥1.00 D SE in the hyperopic eye; strabismus at near and/or distance fixation or history of strabismus surgery; history or present evidence of the visual axis obstruction.

Bilateral amblyopia held to >0.1 logMAR in both eyes after re-testing the child on the comprehensive eye examination wearing full cycloplegic correction, in the presence of a bilateral amblyogenic factor. Amblyogenic factors were defined as bilateral: high hypermetropia ≥4.00 D, myopia ≥6.00 D, astigmatism ≥2.00 D, or history or present evidence of the visual axis obstruction.

Refractive error held to the best corrected VA≤0.1 logMAR after re-testing the child on the comprehensive eye examination wearing full cycloplegic correction in the presence of the amblyogenic factor(s).

Testability was defined for non-testable children. The children who gave answers inconclusively or for whom the VA measurement could not be completed were addressed as non-testable (24) and proceeded as failed the phase. Testability rate was calculated only after the first trial (Phase II).

If the child on the binocular pre-test (Phase I) did not respond at all, the child was labeled as non-cooperative and proceeded as failed the phase. Non-cooperative children were excluded from the testability analysis as the non-cooperative child would respond the same regardless of the VA test administered.

A child was considered healthy if it had monocular VA≤0.1 logMAR in both eyes tested at near and distance and unremarkable ocular status.

### Outcome measures

The outcome measures of the ZAPS study were:

1) Age specific prevalence rates of amblyopia and refractive error;

2) Testability of the ZAPS study protocol;

3) Population-based age-specific normative threshold for determining abnormal monocular VA in preschool children aged 48-54 months using Lea Symbols in lines chart in the City of Zagreb County;

4) Sensitivity and specificity of the near/distance vision test using Lea Symbols in lines chart for near/distance in the City of Zagreb County;

5) Sensitivity and specificity of the ZAPS study protocol using Lea Symbols in lines chart for near and distance in the City of Zagreb County.

### Data extraction for the sensitivity and specificity analysis

Children referred to the Eye Clinic and diagnosed with amblyopia who were labeled as non-cooperative or non-testable at the screening test were excluded from the accuracy analysis.

Sensitivity and specificity analysis for the near and distance vision test separately was performed on the population of patients examined at the Eye Clinic.

Sensitivity and specificity analysis for the ZAPS study protocol was performed using data from the City of Zagreb registration system on VA testing performed prior to school enrollment at health care centers. The VA testing prior to school enrollment is a national health policy conducted by occupational health specialists at health care centers using age-appropriate vision screening test (tumbling E, Landolt C). The screening techniques used by health professionals are not unique in terms of vision test used, approach to the technique of testing, testing distance, pass criterion for the line, pass cut-off criteria for referral to complete ophthalmological examination, or providers’ attentiveness, experience, and training. Children who fail the school-entry screening are referred to pediatric ophthalmologist for comprehensive eye examination.

During the period December 2011-September 2015, the total number of N = 9540 ZAPS screened children performed VA testing prior to school enrollment at health care centers. Additional feedback was provided after the conclusion of the above stated period from the medical centers with pediatric ophthalmology departments in the City of Zagreb County on the number of children who passed the ZAPS study protocol but failed on school-entry screening. The number of false negatives was concluded to be zero as no new case of amblyopia was confirmed for the children who passed the ZAPS protocol but failed on school-entry screening either at the Eye Clinic or at medical centers with pediatric ophthalmology departments in the City of Zagreb County.

### Data analysis

Data are presented with absolute and relative frequencies including 95% confidence intervals (CI). Percentages with respective 95% confidence intervals (CI) were calculated for N = 15 648 subjects in the study, but also as expected values if all eligible N = 16 896 subjects were screened. Data were calculated using MedCalc Statistical Software ver. 15.8 (MedCalc Software bvba, Ostend, Belgium; *http://www.medcalc.org*; 2015) including calculation of sensitivity, specificity, positive predictive value (PPV), and negative predictive value (NPV). Significance level was set to 0.05.

## Results

**Figure 1:**
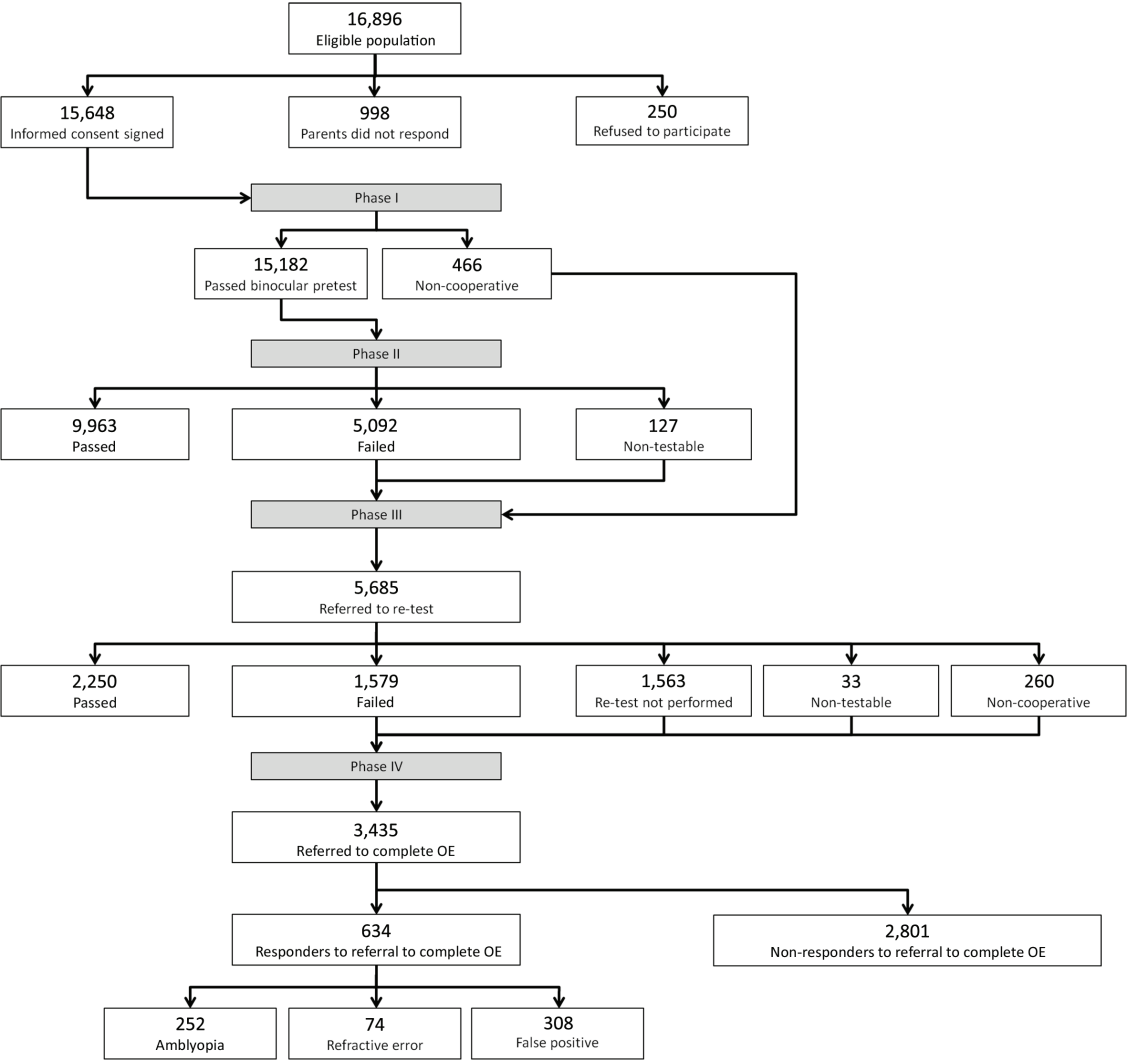
Flow-chart of longitudinal follow-up of all the probands included in the study. OE – ophthalmological examination.

Through September 2011-June 2014, 15 648 children were screened, which determined the attendance rate of 92.61% of the 16 896 children registered in the kindergartens of The City of Zagreb County. Flowchart of longitudinal follow-up of all the probands included in the ZAPS study is presented in Figure 1.

Outcome 1. Age-specific prevalence rates of amblyopia and refractive error. The ZAPS study found the estimated prevalence of amblyopia of 8.08% (Table 1). The estimated prevalence of refractive error was 2.37% (Table 1).

**Table 1:** Characteristics of probands in the prevalence and testability analysis*

						Expected values for the eligible population		
**Proband**	N	/N	%	95% CI	N		%	95% CI
Agreed to participate	15 648	16 896	92.61	92.21-92.99	n/a		n/a	n/a
Parent did not respond	998	16 896	5.91	5.56-6.28	n/a		n/a	n/a
Refused to participate	250	16 896	1.48	1.31-1.67	n/a		n/a	n/a
Amblyopia suspect, after P II	5685	15 648	36.33	35.58-37.09	n/a		n/a	n/a
Amblyopia suspect, after P III	3435	5685	60.42	59.14-61.68	n/a		n/a	n/a
Referred to OE	3435	15 648	21.95	21.31-22.61	3,709		21.95	21.33-22.58
Non-testable after P II	127	15 648	0.81	0.68-0.96	137		0.81	0.69-0.96
Non-testable after P III	33	15 648	0.21	0.15-0.29	36		0.21	0.15-0.29
Responded to referral to OE	634	3435	18.46	17.20-19.79	n/a		n/a	n/a
Amblyopia confirmed	252	634	39.75	36.01-43.61	1,365		8.08	7.68-8.50
RE confirmed	74	634	11.67	9.40-14.40	401		2.37	2.15-2.61
Amblyopia and RE confirmed	326	634	51.42	47.53-55.29	1,766		10.45	10.00-10.92
False positive	308	634	48.58	44.71-52.47	1,669		9.88	9.44-10.34

*N – nominator; /N – denominator; CI – confidence interval; n/a not applicable; P – phase; OE – ophthalmological examination; RE – refractive error.

Outcome 2. Testability of the ZAPS study protocol. Testability rate at the initial examination was 99.19% (Table 1). For 1563 children the re-examination test (Phase III) was not performed due to parental non-compliance. As this subgroup of children failed the initial exam, instructions were given via caregivers in the kindergartens for the child to be referred to complete the ophthalmological examination in the Clinic.

Out of 3435 referred children, 634 responded to complete the ophthalmological examination at the Clinic (Figure 1).

Outcome 3. Population-based age-specific normative threshold for determining abnormal monocular visual acuity in preschool children aged 48-54 months using Lea Symbols in lines chart in the City of Zagreb County. Of 15 648 children in the ZAPS study cohort, 12 213 children (78.04%) passed the screening with the cut-off level ≤0.1 logMAR in both eyes tested at near and distance.

Outcome 4. Diagnostic accuracy of near/distance vision test using Lea Symbols in lines chart for near/distance in the City of Zagreb County, if testing near/distance visual acuity alone. Children referred to the Eye Clinic and diagnosed with amblyopia who were labeled as non-cooperative or non-testable at the screening test (N = 32) were excluded from the accuracy analysis. Near vision test using Lea Symbols in lines chart for near, if performed alone, would have sensitivity of 74.5% and specificity of 43.5% (Table 2). Sensitivity of the distance vision test using Lea Symbols in lines chart for distance, if tested alone, would reach 96.4%, however specificity would be only 11.7% (Table 2).

**Table 2:** Outcome measures. Diagnostic accuracy of near vision test using LEA SYMBOLS® in lines Pediatric Eye Chart for near in the City of Zagreb County, if testing near visual acuity alone (N = 528 children). Diagnostic accuracy of distance vision test using LEA SYMBOLS® in lines Pediatric Eye Chart for distance in the City of Zagreb County, if testing distance visual acuity alone (N = 528 children)

	Screening test – near			Screening test – distance		
	Failed	Passed	Total	Failed	Passed	Total
Amblyopia present	164	56	220*	212	8	220*
Healthy	174	134	308	272	36	308
Total	338	190	528	484	44	528
Sensitivity (95% CI) %	74.5 (68.3-80.2)			96.4 (92.9-98.4)		
Specificity (95% CI) %	43.5 (37.9-49.3)			11.7 (8.3-15.8)		
PPV^†^ (95% CI) %	11.2 (7.9-15.2)			9.4 (6.9- 12.4)		
NPV^†^ (95% CI) %	94.7 (90.9-97.3)			97.1 (88.9-99.7)		

*252 subjects were diagnosed with amblyopia, however, 32 children referred to the Eye Clinic and diagnosed with amblyopia were labeled as non-cooperative or non-testable at the screening test, and were excluded from the diagnostic accuracy analysis of near and distance vision test using LEA SYMBOLS® in lines Pediatric Eye Chart.

†Positive and negative predictive values were calculated with amblyopia estimated prevalence 8.1%. CI – confidence interval; PPV – positive predictive value; NPV – negative predictive value.

Outcome 5. Diagnostic accuracy of the ZAPS study protocol using Lea Symbols in lines chart for near and distance in the City of Zagreb County. The analysis was performed on N = 9228 probands, as children who were referred to the Eye Clinic and diagnosed with amblyopia but labeled as non-cooperative or non-testable at the screening test (N = 32) were excluded from the accuracy analysis of the total number of N = 9540 ZAPS screened children who performed visual acuity testing prior to school enrollment at health care centers. The sensitivity and specificity of the ZAPS study protocol were 100.00% and 96.68%, respectively (Table 3).

**Table 3:** Outcome measures. Diagnostic accuracy of the ZAPS study protocol using LEA SYMBOLS® in lines Pediatric Eye Chart for near in the City of Zagreb County (N = 9508 children)

	Screening test - near and distance		
	Failed	Passed	Total
Amblyopia present	220*	0	220*
Healthy	308	8,980	9,288
Total	528	8,980	9,508
Sensitivity (95% CI) %	100% (98.34% to 100.00%)		
Specificity (95% CI) %	96.68% (96.30% to 97.04%)		
PPV^†^(95% CI) %	72.66% (69.87% to 75.33%)		
NPV^†^(95% CI) %	100% (99.96% to 100.00%)		

*252 subjects were diagnosed with amblyopia, however, 32 children referred to the Eye Clinic and diagnosed with amblyopia were labeled as non-cooperative or non-testable at the screening test, and were excluded from the diagnostic accuracy analysis of near and distance vision test using LEA SYMBOLS® in lines Pediatric Eye Chart.

†Positive and negative predictive values were calculated with amblyopia estimated prevalence 8.1%. ZAPS – Zagreb Amblyopia Preschool Screening; CI – confidence interval; PPV – positive predictive value; NPV – negative predictive value.

## Discussion

The ZAPS study is an extensive investigation of amblyopia prevalence based on the unique, highly standardized screening protocol distinctive for 1) both near and distance VA testing, 2) using testing chart with optotypes in lines and 3) setting VA of ≤0.1 logMAR as the pass cut-off value, administered on a large study-cohort of the 48-54 month-old children.

### Amblyopia and refractive error prevalence

The estimated prevalence of amblyopia among preschool children varies from 0.3% (4) to 5% (5). The ZAPS study found the estimated prevalence of amblyopia to be 8.08%, considerably higher than reported elsewhere. It is speculated that in developing countries, where national screening is not established, higher rates of amblyopia are expected (81). A few studies reported the prevalence rates of visual impairment ranging from 6.3%-31% (45,56,57,60,62). These large discrepancies can be attributed to a wide variety of screening protocols using different screening tests and different designs of the same test, inconsistent criteria for defining amblyopia, amblyogenic factors, refractive errors, and thresholds of age-specific VA normative (4). It is well recognized that optotypes in lines assembled on logMAR principle are the best frame for amblyopia detection (10,34,53,82).

Another important finding of the ZAPS study is the relative preponderance of amblyopia compared to the estimated prevalence of refractive error, suggesting that if significant refractive error is discovered at the age of 4 years it is likely that amblyopia has already been well-established.

### Age-specific normative threshold for determining abnormal monocular VA

This study revealed that 78.04% of 48-54 month-old children reached VA of ≤0.1 logMAR. The Multi-Ethnic Pediatric Eye Disease Study (MEPEDS) group proposed the threshold of 20/40 for defining abnormal monocular VA using HOTV optotypes in children aged 48-59 months (64). The proportion of children achieving the threshold was 99% (64). Compared to our study results and earlier reports on the prevalence of amblyopia ranging 0.8%-10% (30,83-89), we speculate that 20/40 threshold is too low for the 4-year-old age group as this refers only 1% of children to the complete ophthalmological examination, under-representing the number of false negatives who pass the screening but do have some kind of visual impairment. Advocating for more stringent threshold criteria, Vision in Preschoolers (VIP) study group concluded that the threshold levels associated with an increased risk of amblyopia recommended by professional organizations might be exceedingly lenient (90). Our data are in agreement with the study by Leone et al (80), who found the mean VA of 0.26 logMAR (6/11) at <36 months, which improved to 0.1 (6/7.5) at 66 to <72 months using ETDRS HOTV chart.

### Testability

We found an excellent testability rate at the initial examination (99.19%). This is comparable to VIP study results that found Lea Symbols highly testable, with the proportion of 99.5% testable children (67). However, VIP study used a modification of the MassVAT form of the Lea Symbols (91), and not the optotypes in lines presented in the gold standard ETDRS format.

De Becker et al found 95% testability rate using single optotypes of Lea Symbols (58). When Amblyopia Treatment Study (ATS) HOTV protocol was used, testability reached 95% for the 48-54 months aged group (80). It is proposed that HOTV optotypes arranged linearly may be optimal only for children >5 years (80), however, high testability rate of the ZAPS study indicates that optotypes in lines are applicable for children aged 48-54 months if Lea Symbols are used, as they have the contours and meaning that children have already been exposed to and are intellectually easier to perceive and comprehend.

### Diagnostic accuracy

The ZAPS study protocol satisfies the prerequisites of high testability, high sensitivity, and high specificity, which makes it an efficient screening test. The published studies analyzing VA assessment for amblyopia screening in children failed to reach both high testability and high specificity and sensitivity in the same test (22).

The sensitivity and specificity of the ZAPS study protocol were 100.00% and 96.68% respectively. If each of these tests had been performed alone, both sensitivity and specificity would have declined. The former Swedish screening program testing distance VA with HVOT in lines optotypes and the pass level of ≥0.8 for 4-year-old children obtained sensitivity of 86% and specificity of 97% (14). The limit of <0.65 was associated with sensitivity decrease to 70.4% (14). In the study by Bertuzzi et al, VA score set at 0.63 resulted in sensitivity of 78% and specificity of 93% (45). When the cut-off level was raised to 0.8, sensitivity was 96% and specificity 83% (45). Lowering the limit for referral predisposes to a higher number of false negatives. The VIP Study Group set the specificity at 90%, with failure criterion of inability to pass 0.5 line in 4-year-old children and achieved the sensitivity of Lea Symbol test of only 61% (67). When sensitivity was set to 95%, specificity declined to 38%. Overall, the sensitivity of VA tests ranged between 9% (45,60) and 100% (68), and the specificity between 8% (45) and 100% (45,60), however the studies reporting diagnostic accuracy of the screening test used VA testing either for distance or near.

The ZAPS study strengths include a highly standardized study design using optotypes in lines presented in the gold standard ETDRS format as they do not underestimate amblyopia, and population-based results obtained on a large sample size. All screeners were either residents in ophthalmology or ophthalmology specialists. However, the examiner's discretion could be a possible source of bias because their decision for referral was based on the inspection as no further testing was allowed during the screening process. The study limitation could be found in testing the VA twice, first at near followed by distance VA measurement. The larger number of over-referrals due to distance VA testing could be the result of fatigue and limited attention span, as distance acuity measurement followed near acuity testing, and a question is raised if randomization would have reversed the difference. Second, the children who passed the screening were not investigated further with comprehensive eye examination. Hence, we determined the false-negative rate based on the vision screening at school entry, as reported in Methods. Another limitation of the study was a large loss of probands, thus, we could not calculate the prevalence of the sample, but had to present the estimated prevalence, this being the standard procedure in such cases (92,93).

In conclusion, ZAPS study yielded high estimated prevalence rate of amblyopia of 8.1%. To the best of our knowledge, its protocol reached the highest sensitivity and specificity when evaluating diagnostic accuracy of VA tests for screening preschool children aged 48-54 months. Sensitivity of 100% and specificity of 96% advocate that both near and distance VA testing using Lea Symbols in lines chart should be performed in a single session when screening for amblyopia. The chart- and age-specific threshold determining abnormal monocular VA is redefined, and raised to >0.1 logMAR.

The ZAPS study changed the national recommendations for health surveillance in Croatia in favor of VA assessment of 4-year-old children. From June 1st 2015, vision screening of all 4-year-old children performed in ophthalmologists’ practices is introduced as a national health policy. We recommend further systematic examination of the ZAPS study protocol in a longitudinal study design.

## 

Received: October 21, 2015

Accepted: February 23, 2016
